# Invasive Adenosquamous Carcinoma of the Pancreas: Two Case Reports From Tanzania and a Literature Review

**DOI:** 10.1155/crip/3003748

**Published:** 2026-08-20

**Authors:** Hilary Chipongo, Deogratias Mwanjamila, Alex Mashaka, Shafii Ramadhani, Theresia Mushi, Dammy Shimbo, Amos Mwakigonja, Asteria Kimambo

**Affiliations:** ^1^ Department of Anatomical Pathology, Muhimbili University of Health and Allied Sciences, Dar es Salaam, Tanzania, muchs.ac.tz; ^2^ Shree Hindu Mandal Hospital (CBD), Dar es Salaam, Tanzania; ^3^ Department of Anatomy, Physiology and Pathology, University of Dar es Salaam, Mbeya College of Health and Allied Sciences, Mbeya, Tanzania, udsm.ac.tz; ^4^ Department of Anatomical Pathology, Muhimbili National Hospital, Dar es Salaam, Tanzania, mnh.or.tz

**Keywords:** adenosquamous carcinoma, case report, pancreatic cancer, rare pancreatic tumors, sub-Saharan Africa

## Abstract

**Background:**

The prevalence of pancreatic cancer is increasing globally, with over 510,566 new cases reported in the literature in 2022. Adenosquamous carcinoma of the pancreas is a rare histological variant with poor prognosis compared to other exocrine pancreatic cancer subtypes. Little is known about the prevalence of PASC in sub‐Saharan Africa.

**Case Presentation:**

Two cases are presented here: Case 1 reports PASC with invasion of the gallbladder and duodenum, and Case 2 shows early‐onset PASC at an even younger age. Diagnosis was made through clinical evaluation and imaging and confirmed by histological findings.

**Conclusion:**

Due to its similar presentation with pancreatic adenocarcinoma, lack of routine immunohistochemistry in some cases, and limited access to some cross‐sectional imaging, PASC is often misdiagnosed or detected late. These two cases, one with invasion of the duodenum and gallbladder and the other with atypical presentation and hepatic metastasis, highlight the need for a high clinical suspicion and histopathological confirmation in sub‐Saharan Africa.

## 1. Introduction

The prevalence of pancreatic cancer is increasing globally, with a high incidence in developed countries [1]. In 2022, over 510,566 new cases were reported worldwide [1, 2]. The global age‐standardized incidence rate has increased from 5.47 to 5.96 per 100,000 population from 1990 to 2021 [2]. The global burden of pancreatic cancer, measured in disability‐adjusted life years (DALYs), rose from 5.21 million to 11.32 million, and mortality rates showed a similar upward trend, with the number of deaths increasing from approximately 211,613 to 505,752 and the age‐standardized mortality rate (ASMR) rising from 5.655 to 5.948 per 100,000 population [2]. According to recent data in sub‐Saharan Africa, the incidence rate was 0.35 per 100,000 (95% UI, 0.26–0.47), with a death rate of 0.31 (95% UI, 0.23–0.42) and a DALY rate of 14.79 (95% UI, 10.82–19.72) [3]. According to the GLOBOCAN factsheet of 2022, the incidence of pancreatic cancer in Tanzania is approximately 510 cases. Pancreatic adenosquamous carcinoma (PASC) is a relatively rare histological type of pancreatic malignancy, and preoperative diagnosis is difficult because of its rarity [4]. PASC accounts for 1%–4% of all pancreatic cancers [4], with an extremely poor prognosis compared to usual pancreatic adenocarcinoma worldwide [5]. In sub‐Saharan Africa, particularly in countries such as Tanzania, data are scarce, leading to the under‐reporting of this histological subtype. Herein, we report two cases of adenosquamous carcinoma of the pancreas with unique features. Case 1 had contiguous invasion of the gallbladder and duodenum with tumor‐free margins, and Case 2 is notable for early‐onset PASC at age 33 with direct hepatic invasion and portal vein thrombus, which are rarely documented in Africa.

## 2. Materials and Methods

Herein, we report two cases of invasive adenosquamous carcinoma of the pancreas. Histopathological, radiological, and immunohistochemical (IHC) studies were performed and compared with the findings reported in the existing literature. A comprehensive literature search was performed using the PubMed, Web of Science, and Google Scholar databases. Since adenosquamous carcinoma of the pancreas is rare, all available articles relevant to our cases were carefully reviewed, and those relevant to our study were included. The search strategy involved combinations of the terms “pancreatic tumors,” “exocrine tumors,” and “adenosquamous carcinoma.” The inclusion criteria were limited to peer‐reviewed, English‐language articles focusing on human subjects diagnosed with histologically confirmed primary PASC. The exclusion criteria encompassed animal studies, in vitro research, narrative reviews, and letters to the editor lacking original data. After retrieving records, duplicates were automatically removed. Three independent reviewers conducted an initial screening based on titles and abstracts, followed by a detailed full‐text assessment according to the predefined eligibility criteria. Disagreements on study selection were resolved through discussion with a fourth independent reviewer. Additionally, reference lists of all eligible studies were manually examined to identify further pertinent publications. In both cases, formalin‐fixed tissue biopsies were performed, and dehydration, clearing, infiltration with paraffin wax, and embedding were performed to create a tissue block. These FFPE tissue blocks were sliced into thin sections (3 *μ*m) and stained with hematoxylin and eosin (H and E). IHC staining for p63 was performed in Case 2 using a semiautomated approach on an automated staining platform (Ventana Medical Systems). Formalin‐fixed, paraffin‐embedded tissue sections were manually deparaffinized in xylene and rehydrated using graded alcohols to distilled water. Heat‐induced epitope retrieval (HIER) was performed manually using Tris‐EDTA buffer (pH 9.0) in a pressure cooker at 100°C for 30 min. Following antigen retrieval, the slides were transferred to an automated staining platform for immunostaining. Endogenous peroxidase activity and nonspecific protein binding were blocked using reagents supplied within the detection system, according to the manufacturer′s protocol. The sections were incubated with a primary antibody against p63 (clone 4A4) at a dilution of 1:200 for 40 min at room temperature. Immunodetection was performed using a polymer‐based horseradish peroxidase (HRP) detection system (Roche Diagnostics; Catalog Number: Lot 10N06473), according to the manufacturer′s instructions. Signal visualization was achieved using 3,3 ^′^‐diaminobenzidine (DAB). The sections were counterstained with hematoxylin, followed by dehydration, clearing, and coverslipping. Benign prostate tissue was used as the external positive control.

## 3. Case Presentation

### 3.1. Case 1

**Figure 1:**
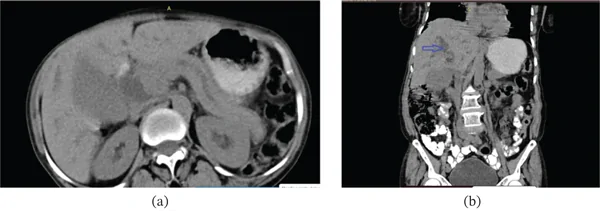
(A) A triphasic CT scan of a hypodense lesion on the head of the pancreas, with bile duct dilation. (B) A coronal view with evidence of hepatomegaly, hepatic hypodense infiltrates (see arrow), and a mass at the head of the pancreas.

**Figure 2:**
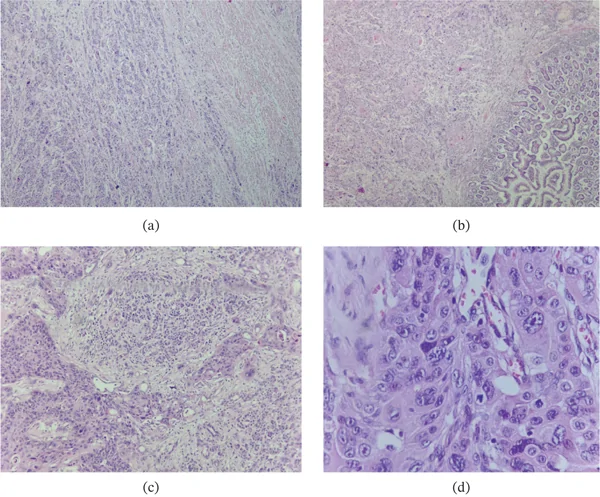
(A–C) Histopathological findings showed pancreatic tissue with a malignant neoplasm composed of atypical ductal structures as well as squamous cell nests, about 60% of the tumor associated with abundant keratin pearls. (D) High‐power view showing numerous atypical mitoses.

A 60‐year‐old woman presented to the Muhimbili National Hospital (MNH) with a history of yellowish discoloration of the eyes for 3 months. Her presenting symptoms were deep yellow urine and pale‐colored stools. She reported other associated symptoms, such as generalized itching of the skin and general body weakness. In terms of social history, the patient had a positive history of alcohol consumption. On examination, the patient was jaundiced, mildly pale, not dyspneic, and had hepatomegaly measuring 20 cm below the right subcostal margin; no tenderness was elicited on palpation. Laboratory investigations revealed a hemoglobin count of 9 g/dL, carbohydrate antigen (CA19‐9) levels of 1200 U/mL, carcinoembryonic antigen (CEA) of 32 ng/mL, and total and direct bilirubin levels of 36 and 12 mg/dL, respectively. A hepatitis panel was performed, which was negative. Triphasic CT revealed a hypodense lesion in the head of the pancreas, bile duct dilation, hepatomegaly with hepatic hypodense infiltrates, and a mass at the head of the pancreas (Figure 1). Intraoperatively, a fungating mass was observed in the head of the pancreas, which was nodular, firm, approximately 4 × 3 × 2.5 cm in size, and largely lodged in the uncinate process, with a distended gall bladder. Histopathological findings revealed pancreatic tissue with a malignant neoplasm composed of atypical ductal structures and squamous cell nests in approximately 60% of the tumor, associated with abundant keratin pearls and numerous atypical mitoses (Figure 2). The gall bladder and duodenal walls were involved, whereas their mucosal surfaces were spared. The excisional margins were free of tumor. The patient was discharged uneventfully after 1 week. She was scheduled for an oncology review at another institute. Two weeks after surgery, chemotherapy was initiated. She received six cycles, each lasting 4 weeks. During the course of the sixth cycle, the patient succumbed. This was about 7 months postsurgery; therefore, she attained a 7‐month survival.

### 3.2. Case 2

**Figure 3:**
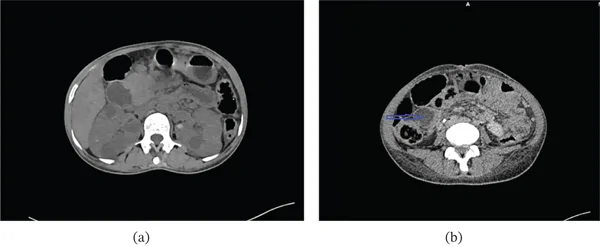
(A) Triphasic CT scan showing pancreatic body mass infiltration into the adjacent gastric wall. (B) Tumor thrombus in the portal vein at the splenic confluence, with direct hepatic invasion of the left lobe and multiple right hepatic metastases (see arrow).

**Figure 4:**
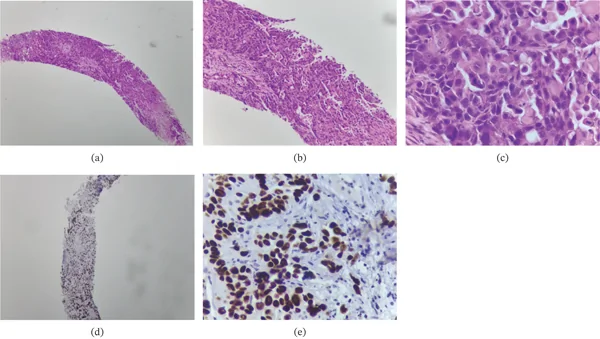
(A, B) ×40 magnification shows a tumor composed of both glandular and squamous components. (C) The glandular component was made up of irregular glands composed of atypical columnar to cuboidal cells, and the squamous component was more than 50% of the tumor, consisting of nests of pleomorphic and hyperchromatic polygonal cells with distinct cell borders and abundant eosinophilic cytoplasm with keratin pearls. (D, E) p63 immunohistochemistry positive for confirmation of adenosquamous carcinoma at low and high power, respectively.

A 33‐year‐old woman was referred from a peripheral hospital to MNH with a complaint of abdominal pain for 6 months, which was accompanied by yellowish discoloration of the eyes. The patient′s social history was unremarkable. On examination, the patient had scleral jaundice, conjunctival paleness, abdominal distention, and hepatomegaly measuring 18 cm below the right subcostal margin. Laboratory investigations showed a hemoglobin level of 8.2 g/dL, CA 19‐9 level of 1300 U/mL, CEA level of 24 ng/mL, creatinine level of 85 *μ*mol/L, and aspartate aminotransferase (AST) and alanine aminotransferase (ALT) levels of 29 and 13 U/L, respectively. Both hepatitis B and C antigens were nonreactive. Triphasic CT revealed a pancreatic mass extending to the body with infiltration into the adjacent gastric wall, a tumor thrombus in the portal vein at the splenic confluence, direct hepatic invasion of the left lobe, and multiple right hepatic metastases (Figure 3). An extended Whipple procedure was performed because the tumor involved the body of the pancreas, and the tissue was sent for histological examination. Histopathological examination revealed that the tumor comprised both glandular and squamous components. Figure 4A,B at ×40 magnification shows a tumor composed of both glandular and squamous components. Figure 4C shows that the glandular component was composed of irregular glands, composed of atypical columnar to cuboidal cells, and the squamous component was more than 50% of the tumor, consisting of nests of pleomorphic and hyperchromatic polygonal cells with distinct cell borders and abundant eosinophilic cytoplasm with keratin pearls. Figure 4D,E shows p63 IHC positivity for the confirmation of adenosquamous carcinoma at low and high power, respectively. The patient was seen in the surgical clinic after 4 weeks. No complications were reported, and the patient was referred for oncology review for possible initiation of chemotherapy. Eight weeks after the surgery, she was seen at the clinic; she was on the second cycle of chemotherapy, and the only complaint was easy fatigue as the patient was still anemic. The last clinic visit was 3 months after the surgery, and the patient was still receiving chemotherapy. No new complaints were observed. Further follow‐up information beyond this period could not be obtained despite efforts to contact the patient.

## 4. Discussion

Adenosquamous carcinoma of the pancreas is a rare pancreatic carcinoma, accounting for approximately 1%–4% of all exocrine pancreatic malignancies [1, 2]. Apart from the pancreas, adenosquamous carcinoma can arise from other parts of the body, including the lungs, esophagus, colon, stomach, salivary glands, and female reproductive system [5]. Adenosquamous carcinoma of the pancreas has a poorer prognosis than adenocarcinoma [4, 5]. In Africa, particularly sub‐Saharan Africa, there are no data on the epidemiology of this rare histological pancreatic malignancy due to resource scarcity and decreased awareness among health practitioners, resulting in under‐recognition of its burden. This is a rare subtype that may clinically present like any other type of pancreatic malignancy, although it carries a poor prognosis and a high mortality rate of more than 95% in the first 5 years [6]. We present two unique cases of adenosquamous carcinoma of the pancreas, one in a young patient with hepatic invasion and the other in an adult with duodenal and gallbladder invasion.

PASC presents similarly to pancreatic adenocarcinoma, with patients diagnosed at a late age, most frequently between 65 and 74 years, with a median age of 70 years [7]. Early‐onset pancreatic cancer has been reported in Africa, particularly in North Africa, where up to 20% of cases are diagnosed at < 50 years of age, which contrasts with the current literature [8]. The reported cases were of common pancreatic cancers, mostly of adenocarcinoma. Rare cases, such as adenosquamous carcinoma, have not been characterized. In Africa, limited cases of PASC have been reported because of its rarity; one reported in northern Africa showed that most of these cases presented at an old age from the 6th decade of life, according to a report by Limaiem et al. [9]. No cases have been reported in sub‐Saharan Africa or low‐income countries. To our knowledge, this is the first report of PASC from East Africa with histopathological confirmation, including that of p63 IHC. However, the absence of molecular testing limits our understanding of the driver mutations in this population. This shows the possibility of having these rare subtypes of pancreatic cancers, which the literature shows to have aggressive behavior even at a younger age. Our case reports a unique case of PASC even at a younger age of 33 years, which indicates that there may be cases presenting at younger ages. In addition, these two cases showed evidence of environmental risk factors, such as alcohol consumption, which may have contributed to the pathogenesis of PASC in Case 1. The absence of identifiable risk factors in Case 2 raises the possibility of underlying germline mutations (e.g., BRCA and PALB2), although genetic testing was unavailable.

Normal pancreatic histology does not have squamous epithelium; therefore, three theories attempt to explain the origin of squamous cells in adenosquamous carcinoma of the pancreas. The first theory (pluripotent stem cell theory) suggests the existence of pancreatic cancer stem cells [8]. The second theory, known as the collision theory, suggests that two histologically distinct tumors develop and integrate independently [10]. The third theory (metaplasia‐differentiation) suggests that the squamous component is the result of the glandular component of a tumor changing its cell type [10]. The metaplastic theory explains that PASC arises from the trans‐differentiation of adenocarcinoma to squamous carcinoma, which explains the clear transition zones between the K8/18+ MUC5AC+ adenocarcinomatous and p63 + K5/14+ squamous components [11]. The transitional zone is characterized by p63 expression in K8/18+ glandular cells and the occurrence of p63 + K5/14+ basally located cells, which differentiate and proliferate to form PASC components [11]. This report is consistent with the metaplastic theory, which shows robust positivity for the p63 marker in PASC cases.

Histologically, adenosquamous carcinoma is diagnosed based on the presence of more than 30% squamous components [9]. This is similar to our cases, in which the squamous component occupied > 50% of the tumor. IHC panel of patients with adenosquamous carcinoma has shown that the cancer is positive for cytokeratin (CK) 5/6, CK 7, and p63 and negative for CK 20, p53, and p16. In our patient who presented at a younger age (Case 2), p63 IHC confirmed the diagnosis of PASC.

There are no classical findings from imaging studies for adenosquamous carcinoma, although many of these neoplasms are characterized by the presence of central tumor necrosis, as well as vascular and nerve encasement on CT scans [12]. These features may provide useful information for characterizing the stage of the disease and predicting prognosis. In our cases, the patients underwent triphasic CT scans, which revealed hypodense lesions on the head of the pancreas with bile duct dilation. Other imaging modalities, such as PET scans and Gallium 67, show increased uptake in adenosquamous carcinoma, unlike pancreatic adenocarcinoma. These investigations were not performed in our case because of limited resources available.

There are no existing guidelines for the treatment of adenosquamous carcinoma of the pancreas; therefore, these patients are treated using the same guidelines as those for adenocarcinoma [12]. Treatment generally includes surgical resection and chemotherapy, depending on the disease stage and the patient′s condition. In our cases, both patients underwent surgical resection, whereby Whipple′s procedure was performed. Following surgical treatment, they were referred to an oncology center for adjuvant chemotherapy, and one died during the course of neoadjuvant chemotherapy after approximately 7 months, while another patient was lost to follow‐up while on neoadjuvant chemotherapy. The literature shows that patients with this subtype have the poorest prognosis compared to those with pancreatic adenocarcinoma, with median overall survival of 8.3 and 15.7 months, respectively [12]. With limited cases, few trials have been conducted involving the use of immunotherapy, such as immune checkpoint inhibitors, providing therapeutic potential when selectively targeting the squamous components of PASC that express PD‐L1.

## 5. Conclusion

PASC is a rare pancreatic exocrine tumor. This article highlights the presence of this deadly subtype in sub‐Saharan Africa, where there is limited data on this aggressive malignancy, and shows that it can even present at younger ages. Hence, in resource‐limited settings, any pancreatic mass with unusual imaging features (e.g., central necrosis and early hepatic invasion) or atypical patient age (< 50 years) should prompt a core biopsy with histopathological assessment for squamous differentiation, and, where possible, p63 IHC.

**Nomenclature**
ALT: alanine aminotransferase
AST: aspartate aminotransferase
BRCA: BReast CAncer gene
CA19‐9: carbohydrate antigen 19‐9
CEA: carcinoembryonic antigen
CK: cytokeratin
CT scan: computed tomography scan
DAB: 3,3‐diaminobenzidine
EDTA: ethylenediaminetetraacetic acid
FFPE: formalin‐fixed paraffin‐embedded
IHC: immunohistochemistry
MNH: Muhimbili National Hospital
PALB2: partner and localizer of BRCA2
PASC: pancreatic adenosquamous carcinoma
PD‐L1: programmed death‐ligand 1
PET: positron emission tomography
TRIS: tris(hydroxymethyl)aminomethane

## Author Contributions

Hilary Chipongo: conceptualization and writing—original draft. Deogratias Mwanjamila: writing—original draft and conceptualization. Alex Mashaka: methodology and investigation. Shafii Ramadhani: methodology and writing—review and editing. Theresia Mushi: writing—review and editing and methodology. Dammy Shimbo: validation and writing—review and editing. Amos Mwakigonja: supervision, writing—review and editing, and visualization. Asteria Kimambo: supervision, writing—review and editing, and writing—original draft.

## Funding

No funding was received for this manuscript.

## Ethics Statement

The authors have nothing to report.

## Consent

Written consent for publication was obtained and, if needed, may be provided to the editor‐in‐chief.

## Conflicts of Interest

The authors declare no conflicts of interest.

## General Statement

*Limitations*. There were a few limitations on these case reports, which included

1. Lack of immunohistochemistry in one case due to patient financial constraints
2. No molecular/genetic or germline genetic testing; these are not performed in our settings
3. Potential selection bias
4. Single institution experience
5. Lost to follow‐up of one of the patients

## Supporting information


**Supporting Information** Additional supporting information can be found online in the Supporting Information section. 

## Data Availability

The data that support the findings of this study are available from the corresponding author upon reasonable request.
