# Supplementary material for: Invasive Adenosquamous Carcinoma of the Pancreas: Two Case Reports From Tanzania and a Literature Review
Source: Case Rep Pathol. 2026 Aug 20;2026:3003748. doi: 10.1155/crip/3003748 (PMC13492051; doi:10.1155/crip/3003748)
Supplement: Supplementary file 1 [file CRIP-2026-3003748-s001.pdf]

CARE Checklist of information to include when writing a case report

CC BY-NC-ND

Topic	Item	Checklist item description	Reported on Line
Title	1	The diagnosis or intervention of primary focus followed by the words "case report"	✓ pg 1
Key Words	2	2 to 5 key words that identify diagnoses or interventions in this case report, including "case report"	✓pg 1
Abstract (no references)	3a	Introduction: What is unique about this case and what does it add to the scientific literature?	✓pg1
	3b	Main symptoms and/or important clinical findings	✓pg 1
	3c	The main diagnoses, therapeutic interventions, and outcomes.	✓pg 1
	3d	Conclusion—What is the main "take-away" lesson(s) from this case?	✓pg 1
Introduction	4	One or two paragraphs summarizing why this case is unique (**may include** references)	✓ pg 2
Patient Information	5a	De-identified patient specific information	✓pg 3
	5b	Primary concerns and symptoms of the patient	✓ pg 4
	5c	Medical, family, and psycho-social history including relevant genetic information	✓pg 4
	5d	Relevant past interventions with outcomes	✓ pg 5
Clinical Findings	6	Describe significant physical examination (PE) and important clinical findings	✓ pg 5
Timeline	7	Historical and current information from this episode of care organized as a timeline	✓ pg 3, 4, 5
Diagnostic Assessment	8a	Diagnostic testing (such as PE, laboratory testing, imaging, surveys)	✓ pg 3, 4, 5
	8b	Diagnostic challenges (such as access to testing, financial, or cultural)	✓pg 3,4,5
	8c	Diagnosis (including other diagnoses considered)	✓pg 3,4,5
	8d	Prognosis (such as staging in oncology) where applicable	✓ pg 3, 4, 5
Therapeutic Intervention	9a	Types of therapeutic intervention (such as pharmacologic, surgical, preventive, self-care)	✓pg 3,4,5
	9b	Administration of therapeutic intervention (such as dosage, strength, duration)	✓pg 3,4,5
	9c	Changes in therapeutic intervention (with rationale)	✓Not applicable
Follow-up and Outcomes	10a	Clinician and patient-assessed outcomes (if available)	✓pg 3,4,5
	10b	Important follow-up diagnostic and other test results	✓pg 3,4,5
	10c	Intervention adherence and tolerability (How was this assessed?)	✓pg 3,4,5
	10d	Adverse and unanticipated events	✓pg 3,4,5
Discussion	11a	A scientific discussion of the strengths AND limitations associated with this case report	✓pg 6,7
	11b	Discussion of the relevant medical literature **with references**	✓pg 6,7
	11c	The scientific rationale for any conclusions (including assessment of possible causes)	✓pg 6,7
	11d	The primary "take-away" lessons of this case report (without references) in a one paragraph conclusion	✓pg 8

**Patient Perspective**	**12**	The patient should share their perspective in one to two paragraphs on the treatment(s) they received . . . .✓patient did not share_
**Informed Consent**	**13**	Did the patient give informed consent? Please provide if requested . . . . .pg 9 . . . . . . **Yes** ✓☐ **No** ☐
